# Experiences of digital exclusion and the impact on health in people living with severe mental illness

**DOI:** 10.3389/fdgth.2022.1004547

**Published:** 2022-11-22

**Authors:** Rachael Middle, Lindsay Welch

**Affiliations:** ^1^ Mental Health and Learning Disability Division 2, Isle of Wight NHS Trust, St Marys Hospital, Newport, Isle of Wight; ^2^ Wessex AHSN and University of Southampton, University of Southampton, Southampton, United Kingdom

**Keywords:** digital, SMI, exclusion, health, inequalities

## Abstract

**Background:**

The covid-19 pandemic has accelerated the use of digital tools within health and social care services. However, for a range of different reasons, across the UK there continue to be people who are digitally excluded. People living with a disability have been identified as being more likely to be digitally excluded and many of these people, including people with severe mental illness (SMI) already experience health inequalities. Therefore, understanding the perceived impact digital exclusion has on health and potential facilitators of increased inclusion is an important area for research. This study had two aims: 1. To understand experiences of digital exclusion and the impact on health in people with SMI. 2. To explore the influences and mechanisms which would increase engagement with digital health tools.

**Methods:**

This was an observational qualitative study, conducting focus groups (with the option of a 1:1 interview for those uncomfortable in groups) with nine people with severe mental illness.

**Results:**

Participant’s responses were themed in to four key areas in relation to digital exclusion and impact on health: 1. Reduced social connectedness, 2. The impact on wider determinants of health 3. Negative perception of self, 4. Disempowerment. Key facilitators for increased engagement with digital tools included, local digital skills support with mental health lived experience involvement in the delivery, digitally engaged social referents, access to digital tools and data, personalised and straightforward digital tools. In addition, increasing health and social care staff’s awareness of digital exclusion was also viewed as important in promoting inclusion.

**Conclusion:**

The research findings suggest that digital inclusion should be viewed as a wider determinant of health. Many of the identified consequences of exclusion are particularly important in relation to mental health and mental health recovery. This research suggests that identifying and addressing digital exclusion should be viewed as a priority for mental health services.

## Background

The covid-19 pandemic has rapidly accelerated the use and adoption of digital technology in health and social care services (1). In addition to video consultations, digital tools in health and care services can also include, the use of apps, wearable devices, smartphones for accessing health information and smart technology (Artificial Intelligence) (2). While there are many potential benefits from increased use of digital tools, including more rapid access to information and personalized care, more control, and empowerment (3), there is also an acknowledged risk of some people being excluded (4).

This is because, despite the pandemic and subsequent increased use of digital tools, 29% of the UK population still has “very low digital engagement (5).” The UK consumer index measures digital engagement through establishing levels of engagement with a range of digital activities. Individuals with “very low digital engagement” are generally not using digital tools such as email. This data is particularly relevant for those with severe mental illness, in light of both the documented benefits of self-management for this population (6), and aspirations for people to manage their own health through the use of digital tools (7). For those who are not able or willing to use digital tools in this way there may be a risk of being digitally excluded and not having the same opportunity (or parity) to utilise the benefits of digital tools for their health.

Digital exclusion could lead to worse health outcomes through both indirect and direct routes (8). Direct routes relate to health services using digital technologies in ways some individuals cannot access and or benefit from. Indirect routes are related to the wider determinants of health, when, for example, housing or employment opportunities, become dependent on digital access routes (8). For people with severe mental illness (SMI), being digitally excluded could exacerbate existing health inequalities. Heath inequalities in people with SMI are driven by factors such as, diagnostic overshadowing (when new symptoms/physical health issues are wrongly attributed to the persons mental health condition), the link between mental illness and poverty, stigma, social isolation and a lack of support to access health and preventative care (9). In England, these inequalities contribute to people with SMI dying on average fifteen to twenty years earlier than the general population. The issue of digital exclusion impacting on people already at increased risk of health inequalities has been described as the “digital inverse care law” with those who most need the benefits that come with digital health tools also being the least likely able to access it (3).

When examining the reasons for digital exclusion, research by Borghouts et al. (10) found that people with SMI can experience additional barriers to engagement with digital tools associated with their mental health condition. For example, symptoms such as fatigue, paranoia and depression can make consistent engagement with digital tools more challenging. This further highlights the importance of exploring and addressing the needs of this population specifically, as they are likely to have different experiences and need a different type of digital support to other groups of people accessing health care.

Berry et al. (11) conducted individual interviews with people living with SMI in 2016 focused on perceptions of self-guided interventions delivered via websites and smartphone apps. Within this research, participants, who reported good levels of digital literacy themselves, expressed concern that there were others who would not have the technology and skills needed to benefit from digital healthcare interventions. This suggests that digital exclusion is an area of importance for this population.

When considering where the gaps in the research are, Helsper (12) concluded that research in to why or how individual’s positions around digital exclusion might change is lacking. This was echoed in a scoping review of digital technology and health inequalities (8). This scoping review concluded that there is a need for further research into what factors influence engagement with digital health technology.

In addition, much of the research previously completed in the area of SMI and digital management of health was conducted prior to the covid-19 pandemic. Therefore, considering digital exclusion and health in the context of the covid-19 pandemic is important due to the potential opportunities to use the findings to influence the delivery of mental health care services going forward. Publications such as, “Build back fairer: The covid-19 marmot review” (13) have helped drive an increased recognition of inequalities and the impact of social determinants on health. The Marmot review provides the impetus for services to “do things differently” and “build back fairer” when resuming services post covid. Therefore, this empirical research focused on two key areas, important in supporting mental health services to “build back fairer”; these are;

To understand experiences of digital exclusion and the impact on health in people with SMI.

To explore the influences and mechanisms which would increase engagement with digital health tools.

## Materials and methods

### Study design

This was an observational qualitative study design. This study utilised focus groups and interviews (14) as a way of eliciting and exploring participants experience of digital exclusion and its impact on health. A qualitative approach was selected as it facilitates an in-depth exploration of participants own behaviour, beliefs and opinions as well as the meaning they attach to their views and experiences (15). Facilitating this was an important aspect of the research, given the hypothesised complexity and nuanced nature of digital exclusion in people living with severe mental illness. In addition, qualitative research is recognised as a valuable method in generating new knowledge in order to enhance evidence based healthcare design (16).

Ethical approval was obtained from University of Southampton, ERGO II number: 66928.There were two members of the research team with lived experience of severe mental illness who acted in an advisory (PPI) capacity. They advised on the research design and participant documents as well as attending the focus groups.

### Sampling and recruitment

Participants were recruited via community organisations supporting people with severe mental illness on the Isle of Wight. The researcher contacted organisations *via* email, attaching the approved study advert and participant information sheet. Two organisations responded to say they had participants who would be interested, and the researcher then followed this up with a face to face discussion to talk through the study.

Ten participants consented to participating in the study, nine participants were recruited via one mental health support group and one participant via a mental health focused housing organisation. However, one participant (recruited *via* the mental health support group) did not attend the focus group and did not leave contact details, so the researcher was unable to follow this up. Therefore, nine participants participated in the study. Of these nine participants, four participants stated that they did not feel comfortable communicating about this topic in a group setting. Therefore, these four participants were offered, and completed, an interview instead of attending the focus groups. Of the remaining five participants, four attended two focus groups and one participant, due to ill health, attended only the first focus group.

The eligibility criteria were:

**Table:** 

Inclusion	Exclusion
Severe mental illness- “psychological problems that are so debilitating that ability to engage in functional and occupational activities is severely impaired” (9)	Mental illness which does not severely impact on ability to engage in functional and occupational activities.
Self reported digital exclusion and rating themselves with a score of 1–7 on a digital inclusion scale (17)	Self-rating of 8 (confident) or 9 (expert) on a digital inclusion scale (17)
Over 18 Years Of Age	People with moderate/severe/profound cognitive impairment were not included in the study due to the likely impact of this level of cognitive impairment on the use of digital tools.

### Screening questionnaire

The digital inclusion scale (17) was used as a tool for screening level and type of digital exclusion (Supplementary Appendix A). This scale was chosen due to its simple accessible format and the ability to differentiate between the different levels and types of exclusion. Mental health diagnosis and socio-demographic information (age, gender, education level and employment status) was also collected. This information was collected as it is known to influence digital use.

### Procedure

Two focus groups were facilitated by the lead researcher. Focus groups were selected primarily due to findings that focus groups can facilitate the sharing of sincere attitudes and beliefs due to their less formal nature, compared to one-to-one interviews (18). The decision to run two focus groups was made to ensure that participants had long enough to discuss their views but also to mitigate against the potential impact of fatigue. All participants were made aware of the role of the researcher, (employed by Isle of Wight NHS Trust within a mental health clinical improvement role). They were also made aware that this research was conducted through a research initiation award with “Wessex Applied Research Collaboration” with support from University of Southampton.

Each focus group lasted approximately 45 minutes and was held at the community location, in a private space, that participants would usually meet to attend their mental health support group. Five participants attended the first group and four participants attended the second, one participant was not able to attend the second due to experiencing a deterioration in his mental health. Four participants met with the researcher to complete a 1:1 interview, these were offered to be inclusive of those participants who stated that they were not comfortable sharing their experiences in a group setting. The interviews lasted between 10 and 20 minutes and were completed at the location of the community support group (*n* = 3) and at the persons home address (*n* = 1).

The focus groups and interviews followed a topic guide (Supplementary Appendix B) which was developed for the study based on a review of the literature (10, 11, 12, 19).The topic guide was also discussed and agreed with the two members of the research team with lived experience of SMI. The first focus group asked participants about their experiences of digital exclusion and how digital exclusion has impacted on their health. The second focus group asked participants to discuss what might have to change in their lives to enable increased use of digital tools and how any support needed in relation to this might be delivered.

Prior to the focus group and interviews starting the lead researcher talked to participants and explained what is meant by the term digital tools and what this can include so all participants were interpreting this term in the same way.

### Data analysis

The focus groups and interviews were audio-recorded and transcribed verbatim by the lead author. Thematic analysis, as a six phased method (20), was used as the method for examining the perspectives of the research participants and identifying themes. The data was viewed from an interpretivist standpoint (21).

Following audio transcription, the lead author listened back again to the recording while reading the transcript in order to familiarise themselves with the data. Initial coding was then completed, every line of the data was coded. While acknowledging the active role of the researcher and their epistemological position, social constructionism (21), an inductive approach to coding was taken, driven by the data. The next three steps involved theming the codes, then refining and naming the codes. Several thematic maps were generated as part of this refining process. Peer debriefing (22) was utilised throughout the coding and theming steps, with three members of the research team (NC, RE, LW). Once the themes were identified and named, member checking, checking the preliminary researcher findings and interpretations with the research participants, was completed with eight of the participants in order to generate the final themes and thematic map. Peer debriefing and member checking are recommended steps in achieving trustworthiness when using thematic analysis (22). These steps were also important elements of the process of engaging in reflexivity (23).

## Results

Participants’ age ranged from 32 to 73 years (*M* = 54, SD = 14.73). As shown in Table 1, there were slightly more female participants (*n* = 5;55%) than males. The majority of the sample had a diagnosis of anxiety and/ or depression (*n* = 7; 78%), and no participants were currently working, two participants were employed but signed off sick. The participant’s self-reported level of digital inclusion ranged from “never have never will” (*n* = 1) through to “task specific” (*n* = 3).

**Table 1:** Participant demographic information.

Demographic Information	Frequency	Percentage
Gender		
Male	4	44%
Female	5	56%
Primary Mental health diagnosis		
Anxiety	4	44%
Depression	2	22%
Panic disorder	1	11%
Hearing voices	1	11%
Borderline personality disorder	1	11%
Employment status		
Sick leave	1	11%
Retired	3	33%
Volunteer	1	11%
Unemployed	3	33%
Student	1	11%
Level and type of digital exclusion		
1-never have, never will	1	11%
2-was online but no longer	1	11%
3-willing and unable	1	11%
4-reluctantly online	2	22%
6-task specific	4	44%
7-basic digital skills	0	0
8-confident	0	0
9-expert	0	0
Additional medical information provided		
Hearing difficulties	2	22%
Autism	2	22%

**Figure 1:**
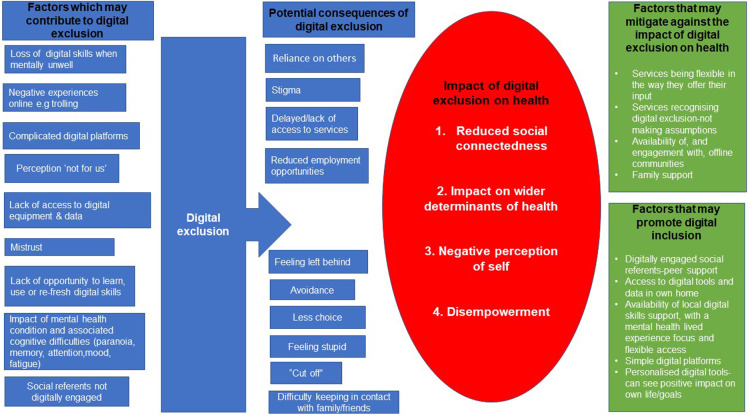
Thematic map.

In relation to digital exclusion and health four key themes were identified: social connectedness, wider determinants of health, negative perception of self and disempowerment. The key themes as well as participants identified factors contributing to digital exclusion and factors which may alleviate the impact of exclusion/promote inclusion are presented in a thematic map (Figure 1).

The key themes and additional factors are elaborated on below, with quotes from the participants. Participants who contributed via the focus groups are recorded as participant numbers 1–6. Participants who inputted via 1:1 interview are recorded as participant letters A-D.

### Theme 1. Digital exclusion impacts on social connectedness

#### Family

Participants all discussed the role and importance of digital tools in connecting with others, in particular family, with one participant explaining how crucial this is:

“*during two spells in hospital when I had to put things in a bag umm you know like some spare socks and boxers and things like that to get in the ambulance, I would always make sure I had my phone charger with me … even when so unwell and feeling this is the end of the road now, that went into the bag, because its communication isn’t it with your famil*y …” (Participant 5)

One participant discussed how even experiencing some digital exclusion (not using social media) can make maintaining this connection more challenging:

“*Umm it makes some communications difficult, like my entire family are on Facebook and I’m not and a lot of them don’t live over here so it’s harder to keep in touch coz no one wants to go through the effort of a phone call these days”* (Participant A)

#### Local community

Another participant discussed that not being on social media, makes it more difficult to hear about community events:

“*…* *it’s good for messages or if you see there’s an event, good for that side of it, that’s the sort of things you might miss”* (Participant D)

There was agreement across participants that digital inclusion can be important in reducing social isolation and feeling “cut off”. Participants expressed that being enabled to have the ability and choice to use digital tools in their own home is an important part of feeling connected to others.

However, in contrast, there was also concern that the accelerated use of digital tools in society is in part to blame for perceived loss of local community infrastructure:

“*I feel it’s a bad thing sometimes* *…* *I think you know the high streets disappearing, lots of people are losing their jobs because of the internet* *…”* (Participant 1)

#### Society

There was a consensus that being digitally excluded can lead to a feeling of disconnect with wider society:

“*I think a lot of us we’re scared to use technology but at the same time we feel we’re being left behind because we’re not in that group if you like”* (Participant 1)

However, there was also concern that with increased digital inclusion comes reduced opportunities for face to face contact in society and that this has a negative impact on people with mental illness:

“*And I know everything seems to be going over to this but I can’t really see how it would work really umm it just can’t because we’re human beings not a machine and we require reassurance all the time, especially if we’ve got mental health problems, that’s the whole idea of mental health is talking to some body and getting some feedback” (*Participant 6)

### Peer support and offline communities

The group placed value on connecting with offline communities, such as their mental health support group:

“*… Its why this groups so successful, it’s not technology its people talking which makes you feel better, it’s the personal touch if you know what I mean, you can bounce off each other, you suffer like I suffer and all that but you don’t get that, not on the internet I don’t think anyway”* (Participant 1)

There also appeared to be a feeling of increased connection from a shared group identity around digital exclusion:

“*… We’re not expected to do something on the computers or on our phones we can just sit with each other”* (Participant 2)

Nearly all participants felt that there was a role in this connection, with others with lived experience of severe mental illness, acting as a potential bridge to increased digital inclusion:

“*… I don’t have many (friends) so their opinions are very valued to me and a lot of them have mental health disorders too, so you know they’ve been through a lot of the same things as me, and I think if they have positive experiences with it (digital tools) maybe I can”* (Participant A)

This also applied to any potential training courses offered around use of digital tools:

“*Ideally this person doing the 1:1 will have had depression not just know about it but actually lived it, unless you’ve actually lived it you don’t realise how crippling and restrictive it can be, you know”* (Participant 1)

“you’d feel comfortable with people a bit more like yourself that, you know, know what it’s like” (Participant 4)

### Theme 2. Digital exclusion and social determinants of health

Participants discussed the affect digital exclusion can have on some of the social, economic and environmental factors which influence physical and mental health and wellbeing.

#### Employment and benefits

For one participant, not having adequate level of digital skills and appropriate digital equipment impacts on her employment options:

“*… the technology that I’ve got moving forward isn’t enough* *…* *so yeah in that way me moving forward I need that really”* (Participant D)

Limited employment options then exacerbate her financial stress:

“*I get £57 per week, I’m struggling, (becoming upset) … sorry”*

The participant explained that she has recently had an offer of help from someone in the local community to support her to learn the digital skills she needs and considers this help to be “*a lifeline”*.

Another participant explained the challenges associated with applying for benefits without the digital skills needed to use email and how much longer this takes when he has to ask his employer to assist with this.

#### Housing

One participant explained that not having the digital skills needed to access online services meant that there has been delays in addressing issues with his housing situation, negatively impacting on his mental wellbeing:

“*I felt err, nothing, I was getting nowhere; nothing was changing and the problem wasn’t going away and I was stuck if you like”* (Participant 5)

#### Access to services

Participants discussed that being digitally excluded has altered the type of health input available to them:

“*I was once trying for some treatment over the phone, like a consultation you know, health wise, over the phone, not over the phone over the internet and I couldn’t cope with it at all, just couldn’t cope with it”* (Participant 5)

Participants also discussed how the combination of inaccessible digital platforms and cognitive difficulties associated with their mental health condition (e.g., memory difficulties) can create barriers to access. This has resulted in participants not being able to access services they have needed.

Some participants reported that covid-19 had made access to health services more difficult due to increased use of digital platforms:

“*And I understand about covid but it is a bit like since covid it does feel a bit like the doctors and mental health services have all gone there you go do it online don’t bother us”* (Participant 4)

However, two participants, with hearing difficulties, discussed the potential for digital tools, such as the use of text messaging and emailing, to support improved access to services.

“*I used to have a support worker but I don’t anymore, last month it took me two weeks to phone up the GP surgery for hearing aid batteries, but I did speak to them and explained how anxious I get phoning and they have for the first time ever, I am 42 now and have seen them since I was 4, they have given me an email address that I can contact them on”* (Participant 2)

Offering email as a form of contact, is an example of the potential positive impact the acceleration in adoption and use of digital tools during the covid-19 pandemic can have on health care accessibility. However, participants emphasised that in their experience services are not consistently adopting the flexible and individualised approach needed to facilitate digital inclusion.

### Theme 3 digital exclusion can contribute to a negative perception of self

#### Stigma

Most participants discussed awareness and experience of stigma associated with having a mental illness:

“*I think the mental health is the Cinderella because people tend to think that we’re useless don’t they, that we shouldn’t be a burden to society”* (Participant 6)

In addition to the stigma associated with mental illness, participants also described experiencing a second layer of stigma associated with digital exclusion:

“*Maybe there’s a bit of a stigma as well where you don’t really want to say you don’t understand because you feel stupid you know”* (Participant 1)

“*… everybody’s on social media using apps and things like that aren’t they? It’s literally the simplest thing and it’s taken for granted mostly, but when I tell people that I don’t have social media they kind of look at me as if I’ve got two heads or something”* (Participant A)

The impact of this perceived stigma appears to be reinforced by others assuming a certain level of digital inclusion. Participants all expressed experiencing services demonstrating a lack of awareness around digital exclusion:

“*I think there’s far too much assumption by, umm the support, the health support and things that we can all just automatically use technology. I think that seems like a given now, well you just go online* *…* *and I’m afraid they have to realise that isn’t the case”* (Participant 5)

“*They take it for granted* *…* *they just usually say oh just go on the computer, I haven’t got one, oh you know, just go on the internet”* (Participant 2)

#### Self-esteem

Nearly all participants commented that not having the ability/access to utilise digital tools has impacted on the way they feel about themselves:

“*I suppose it makes you feel vulnerable using technology that you’re not sort of fully or you don’t understand it fully”* (Participant 6)

“*Frustrating, feel as though I’m backward, horrible”* (Participant C)

However, for one participant, coming offline, after negative experiences with online support forums, social media (trolling) and search engines on health anxiety, can have a positive impact:

“*it’s good to have those things limited so I’m exposed to triggers as little as possible* *…* *if I’m having a particularly bad mental health day I have to disable the internet on my phone so the temptations not there to make myself worse”* (Participant A)

#### Social identity

The participants also discussed how not using digital tools can influence how they perceive themselves to be viewed by others and their place in society:

“*they get frustrated don’t they”* (Participant 2)

“*It’s a bit embarrassing as well though isn’t it? like you’re out of touch, don’t you use these things?”* (Participant 2)

“*you’re sorta left behind aren’t you”* (Participant 1)

### Theme 4. Digital exclusion contributing to a feeling of disempowerment

#### Self- efficacy

Participants discussed previous attempts to engage with digital tools which had not been successful. They described how this had negatively affected their wellbeing and their motivation to engage with digital tools again in the future. This lack of belief and motivation (self efficacy) in their ability to engage with digital tools, appeared to contribute to a general sense of disempowerment

“*The very fact that you struggle with technology makes your anxiety even worse in a way, because you’re frustrated that you can’t do it so you tend to avoid it because you know you’re going to get annoyed with it”* (Participant 1)

“*You just find yourself saying I can’t do it* *…”* (Participant 5)

#### Self-determination

Participants agreed that digital exclusion can result in a loss of choice and control when accessing health services.

“*It’s a backward step isn’t it really* *…* *it seems to limit your choices then doesn’t it of what sort of help you can get”* (Participant 5)

Some participants felt that their digital exclusion was driven by a lack of opportunity to learn how to use digital tools:

“*We weren’t taught it* *…* *we’ve had to learn it bit by bit and perhaps if you do a job which you have to use a computer there, but otherwise you’re left to your own devices aren’t you”* (Participant 6)

All recognised that having control and choice over your own level of digital inclusion was important associating digital competence with “power” (Participant 4)

#### Reliance on others

Many participants highlighted that not having digital skills or access to appropriate digital equipment leads to increased reliance on others, particularly family:

“*my phone is my brothers ex contract phone, he got a new contract and he gives me his old one, otherwise I wouldn’t have one, no way”* (Participant 2)

*“my mum helps me with it”* (Participant B)

“*… you have to ask people all the time you know”* (Participant 1)

Sometimes this can be challenging if the person does not have family/friends with the digital skills needed to support:

“*I don’t have anyone else to ask to help me, coz my mum bless her she’s useless with technology, I have to help her and I don’t know much* *…* *but so then sometimes you end up giving up because you’re just like, oh no its too much”* (Participant 4)

Two participants discussed the positive impact good digital skills support can have, associating their new skills with a feeling of increased independence.

“*I’ve actually got the banking app, only coz my bank were lovely I went in and explained everything and he actually sat down downloaded it with me, it worked, miracle, and then showed me how to use it and everything so I know how to do that now, I’m confident to do that, but that’s him taking time out to show me, you don’t always find someone”* (Participant 2)

This was in contrast to other experiences of digital support. These were described as disempowering and were characterised by a “one size fits all”, rushed approach with digital support being “done to them” rather than with them.

## Discussion

The aim of this study was to view the experiences of digital exclusion in people with SMI in the context of health as well as the mechanisms and influences of increased digital inclusion. This study found that digital exclusion can impact on the health of people with SMI both directly and indirectly, through the wider determinants of health. Digital inclusion could be increased through understanding and addressing the complex relationship between digital skills, access to equipment and the role of local community and digitally engaged peers.

### Digital exclusion and health

The data and themes from this study highlight the broad and complex relationship between digital exclusion and health. This relationship is particularly pertinent when viewed from a mental health recovery perspective (24). Three of the themes identified in this study, relating to social connectedness, perception of self and empowerment, are viewed as important components of mental health recovery. The acronym CHIME has been used to describe five components of personal (mental health) recovery, connectedness, hope and optimism about the future, identity, meaning in life and empowerment (24). The positive impact of belonging and meaningful connections on wellbeing and mental health in people with SMI was also highlighted by Barut et al. (25).

Participants discussed the value of their links with, and support from, offline communities, which may provide some of this belonging and connectedness and mitigate against the loss of social connectedness arising from digital exclusion. However, even with these offline connections, participants discussed difficulties forming or maintaining other connections when experiencing digital exclusion. The potential for digital exclusion to lead to a feeling of disconnect with wider society was evident through participants conveying a sense of “us and them” when discussing digital use.

In addition, there were concerns from participants that accelerated use of digital tools across society was leading to a loss of community infrastructure. The presence of local community infrastructure is known to be important in fostering a sense of belonging in the community. This has been found to be important in relation to promoting good health and mitigating against conditions such as, stress, depression, addiction, and chronic physical ill-health (26).

This experience of accelerated digital use in society and perceived loss of local community infrastructure, likely exacerbated by restrictions on social contact and mobility during covid-19, may be leading to a sense of mistrust and apprehension about engaging with digital tools. The areas most likely to have seen the loss of community infrastructure are also areas with highest levels of social disadvantage, with southern coastal towns (this study was located in a Southern Coastal town) highlighted as being more likely to have areas of intense social deprivation compared with other parts of the UK (27). This is relevant to this research, given the location of the participants and the relationship between mental illness and poverty (28).

Participants discussed the feeling of stigma that comes from their mental health diagnosis. They also reported an additional layer of stigma attributed to their digital exclusion. The anticipation of discrimination associated with stigma can lead people to use strategies of avoidance and concealment (29). This links with experiences relating to digital exclusion stigma described by participants in this study. For example, the participants talked of being reluctant to tell people they struggle with using technology and avoiding using it. This perceived stigma also appeared to create or exacerbate negative feelings about themselves, arising from not being able to access/use digital tools and the subsequent increased reliance on other people. This is important when considering impact on health, as a good quality of life in people with mental health problems has been found to be characterised by elements including control, autonomy and a positive self-perception (30). In contrast low control is associated with poorer health outcomes (13) and regular experiences of enacted or perceived stigma and self-stigmatising has been found to have a negative impact on areas including engagement with services and self-management (31).

Digital inclusion is one aspect of life, and there are other influences on positive self-perception, belonging and control. However, participants did equate digital skill/access with concepts such as “independence” and “power” while reporting that digital exclusion can result in a loss of choice and increased reliance on others. Therefore, supporting people to be in a position to make informed decisions about their level of digital engagement through addressing barriers could have a positive impact on perceived quality of life and on health more broadly.

Health is also influenced by social determinants and participants in this study discussed the ways in which being digitally excluded can negatively impact on this area. For example, they reported experiencing, delayed, altered or a lack of access to housing, employment, benefits and health services. This supports the model put forward by Davies et al. (3) outlining the ways that digital exclusion both directly and indirectly impacts on health inequalities and arguing for the need for digital exclusion to be recognised as a social determinant of health.

### Mechanisms and influences of increased digital inclusion

When discussing factors that may increase engagement with digital tools, participants echoed the need to consider factors identified in previous research such as cognitive difficulties associated with their mental health condition, financial barriers and the complexity of the digital tool (10, 11, 19). In addition to these several other factors were identified, including the importance of access within the local community and the presence and influence of digitally engaged peers.

The experiences and thoughts expressed by participants in this study indicated a strong feeling of connection with others experiencing severe mental illness. This connection appeared key to influencing their possible future engagement with digital tools. Participants talked about the role of others with mental illness delivering digital skills support and the benefits of this in facilitating accessible digital skills support where mental health related reasonable adjustments are built into the design and delivery. Participants also talked about the role of others with mental illness, with higher levels of digital engagement, sharing their positive experiences as being a motivator to increasing their own level of digital engagement.

The importance of social referents supports the findings of Helsper (12) who discussed the importance of social identity and group comparisons in digital inequalities, with some individuals possibly viewing their digital exclusion as “the norm” for them and those they view as their referents. This may then foster a feeling of acceptance of their current situation, reducing the individual’s motivation to change. Mental health peer support is a recognised role in NHS mental health services, and there is evidence to support the positive impact this role can have on levels of hope, empowerment and quality of life (32). The provision of peer support in the area of mental health and digital skills support could be beneficial in building on the value of shared social identity as a way of increasing digital inclusion.

Participants stated that health and care professionals do not ask about their level of digital inclusion and as a result often make assumptions of digital access and competence. This may perpetuate a perception of stigma around digital exclusion and suggests that there is under recognition of digital exclusion within services. Recognising and identifying digital exclusion needs is an essential first step in increasing digital engagement.

Participants gave examples of how health services using digital tools flexibly, based on an individuals need, could have a positive impact on access to and engagement with services. For example, the use of text messaging to confirm appointments and or communicate basic health information. However, their experiences suggest that often the availability and implementation of digital tools can be too rigid meaning that the benefits of digital are not accessible to all.

Participants valued local community and connection with others, and described a perception that society's increased use of digital tools/services has contributed to a loss of local community infrastructure. Participants also reported they have not had the opportunity to learn and or maintain digital skills through education or employment. Therefore, local community based digital support services may provide a positive way of reinvigorating local highstreets and fostering a sense of belonging for local people. This could also provide opportunity to learn, refresh and maintain digital skills. The potential for this type of support to have a positive impact was highlighted by one participant who had received beneficial support characterised by “time” and “patience” from a digital support service at a bank. However, choice and control over how and where they use digital tools was seen as important with participants also discussing the need for this to be available in their own homes.

### Limitations

This study was a small qualitative study, exploring the experiences of nine people with severe mental illness on the Isle of Wight. The small number of participants, somewhat limits the generalisation of findings across the whole population. However, there was a range of ages represented and mix of males and females. In addition, the findings from this study are in line with findings from other research carried out in the area of mental health and digital exclusion which adds to the validity of the findings.

Nearly all participants were recruited from the same mental health support group. This possibly enabled more open interaction within the focus groups as participants were familiar with each other. However, the homogenous geographical location may have an influence on the experiences of digital exclusion for this group of people.

Some socio-demographic data was collected as part of this research (age, gender, employment status and education level). However, data around housing status and ethnicity was not collected.

This study looked specifically at the experiences of people digitally excluded, therefore it is not possible to draw conclusions relating to which factors in particular lead to some people with severe mental illness being digitally excluded and others not. However, it was noted during this study that none of the participants were currently in full or part time employment (although one person was on long term sick leave). Future research could look to establish the factors which influence digital engagement most within a population.

Further research would also be beneficial in evaluating the delivery of digital support, addressing areas raised in this research, and its impact on an individual’s health. This is important in understanding the effectiveness of models for addressing digital exclusion and the impact on health.

## Conclusion

This study identified four overarching themes that relate to digital exclusion and health in people with severe mental illness: social connectedness, wider determinants of health, negative perception of self and disempowerment. These themes highlight the relationship between digital exclusion and health and support arguments that digital inclusion should be viewed as a social determinant of health. These themes appear particularly pertinent when viewed in the context of mental health and mental health recovery.

This suggests, that for mental health services, enabling informed choice around digital engagement should be viewed as an important part of their role in promoting good health. Addressing identified barriers to digital inclusion, through considering facilitators to engagement such as; access to local digital skills support with a mental health peer support component, awareness of digital exclusion amongst mental health staff and access to equipment and data. Furthermore, flexible and individualised use of digital tools by services should be viewed as a priority for mental health services.

## Data Availability

The raw data supporting the conclusions of this article will be made available by the authors, without undue reservation.

## Data sharing

Access to the underpinning research data set can be obtained by contacting the lead author of the paper.
