# Supplementary material for: Experiences of digital exclusion and the impact on health in people living with severe mental illness
Source: Front Digit Health. 2022 Nov 22;4:1004547. doi: 10.3389/fdgth.2022.1004547 (PMC9722951; doi:10.3389/fdgth.2022.1004547)

**Digital inclusion scale (UK Government 2014)**

**Please circle where you are with using digital tools to support your health on the scale:**


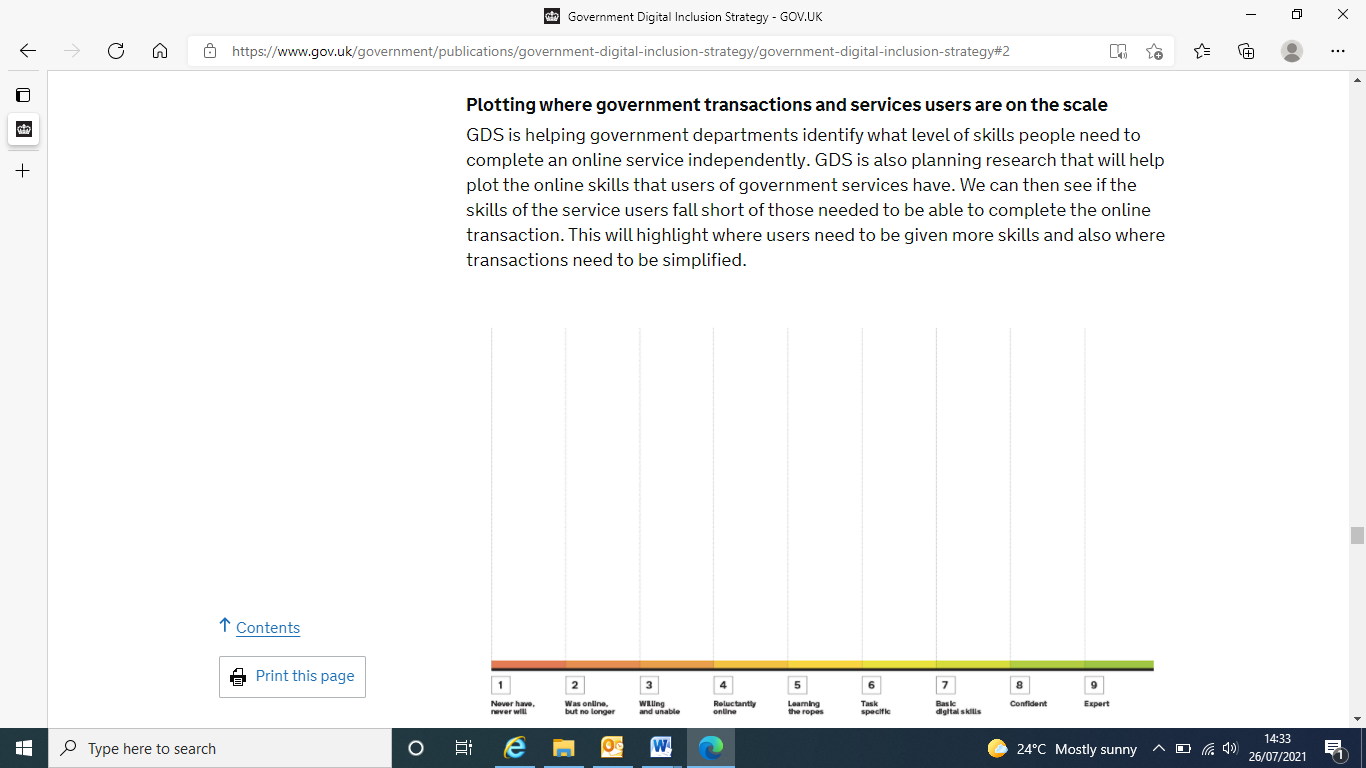

Supplement: Supplementary file 1 [file Datasheet1.docx]
