# Supplementary material for: Experiences of digital exclusion and the impact on health in people living with severe mental illness
Source: Front Digit Health. 2022 Nov 22;4:1004547. doi: 10.3389/fdgth.2022.1004547 (PMC9722951; doi:10.3389/fdgth.2022.1004547)
Supplement: Supplementary file 2 [file Datasheet2.docx]

**Topic Guide**

The purpose of the focus group:

The purpose of this focus group is to hear about different people’s experiences and feelings about digital exclusion and health. Focus groups are used a lot in research to help researchers understand a particular topic. With the permission of people in the group the focus groups will be audio recorded so the researcher can make a transcription of what was discussed.

The group will be asked to discuss the questions below. There are no right or wrong answers, people in the group might have different opinions, this is ok. People in the group can say as much or as little as they want to. I ask that everyone in the group keeps the things discussed in the session confidential.

I hope you enjoy the sessions and feel you can be open and honest with your experiences and challenges in using digital tools to support your health.

Focus group 1:

- What have been your experiences of managing your health and being digitally excluded?
- How has being digitally excluded impacted on your health?

Focus group 2:

- What would have to change in your life for you to think about going online or using digital tools more to support your health?
- What would be the most comfortable way for you to learn to use digital technology to support your health?
- How might the support you need be delivered?
